# Supplementary material for: Genetic Diversity and Spatiotemporal Dynamics of Chikungunya Infections in Mexico during the Outbreak of 2014–2016
Source: Viruses. 2021 Dec 31;14(1):70. doi: 10.3390/v14010070 (PMC8779743; doi:10.3390/v14010070)
Supplement: Supplementary file 1 [file viruses-14-00070-s001.zip › 1477727_Supplement.pdf]

## Table S1

### Reaction Mix

| Reagents | Volumen |
|---|---|
| **RNA** | 5 µl |
| **One step Buffer 2x** | 10 µl |
| **Primer Mix** | 2 µl |
| **Enzyme Mix** | 0.2 µl |
| **PCR grade water** | 2. 8 µl |
| **Total** | 20 µl |

### Thermal cycler program

- 42°C by 60 min.
- 94°C by 3 min.
- 35 cycles:
    - 94 ° C by 30 seg.
    - 60 ° C by 30 seg.
    - 68 ° C by 45 seg.
- 68 ° C by 5 min.
- Hold 4 ° C

| Primer | Sequence |
|---|---|
| Chik_NSP3 Forward | 5'- TCGTCGGCAGCGTCAGATGTGTATAAGAGACAGCCCGTCCCGTCAGACCTG -3' |
| Chik_NSP3 Reverse | 5'- GTCTCGTGGGCTCGGAGATGTGTATAAGAGACAGCTCGTCGTCCGTGTCTGAGC -3' |
| Chik_E1 Forward | 5'- TCGTCGGCAGCGTCAGATGTGTATAAGAGACAGCAGGGCTCATACCGCATCCG -3' |
| Chik_E1 Reverse | 5'- GTCTCGTGGGCTCGGAGATGTGTATAAGAGACAGGGCATGTTCCCTACGGCG -3' |
| Chik_E2 Forward | 5'- TCGTCGGCAGCGTCAGATGTGTATAAGAGACAGCATGCCCCCAGACACCCC -3' |
| Chik_E2 Reverse | 5'- GTCTCGTGGGCTCGGAGATGTGTATAAGAGACAGCCCCACGTGACCTCGAGC -3' |

**Table S1 supplement.** PCR conditions and primers used for NSP3, E1 and E2 partial gene amplification.

## Table S2

### Adapters

| | |
|---|---|
| **Fordward** | 5' TCGTCGGCAGCGTCAGATGTGTATAAGAGACAG-[locus specific sequence] |
| **Reverse** | 5' GTCTCGTGGGCTCGGAGATGTGTATAAGAGACAG-[locus specific sequence] |

### Index

| Library | INDEX | Library | INDEX |
|---|---|---|---|
| CHIKV Pool 1 | S517/N701 | CHIKV Pool 13 | S517/N704 |
| CHIKV Pool 2 | S502/N701 | CHIKV Pool 14 | S502/N704 |
| CHIKV Pool 3 | S503/N701 | CHIKV Pool 15 | S503/N704 |
| CHIKV Pool 4 | S504/N701 | CHIKV Pool 16 | S504/N704 |
| CHIKV Pool 5 | S517/N702 | CHIKV Pool 17 | S517/N705 |
| CHIKV Pool 6 | S502/N702 | CHIKV Pool 18 | S502/N705 |
| CHIKV Pool 7 | S503/N702 | CHIKV Pool 19 | S503/N705 |
| CHIKV Pool 8 | S504/N702 | CHIKV Pool 20 | S504/N705 |
| CHIKV Pool 9 | S517/N703 | CHIKV Pool 21 | S517/N706 |
| CHIKV Pool 10 | S502/N703 | CHIKV Pool 22 | S502/N706 |
| CHIKV Pool 11 | S503/N703 | CHIKV Pool 23 | S503/N706 |
| CHIKV Pool 12 | S504/N703 | CHIKV Pool 24 | S504/N706 |

**Table S2.** Oligonucleotide (primers) sequences of Illumina adapters and indexes used in library prep kits.

## Table S3

| Library | Location | Raw reads | After filtering | After trimming | After paired | Clusters | Clusters at Effective Sample Depth 95% | Secuences after collapse |
|---|---|---|---|---|---|---|---|---|
| 1 | Baja California | 242552 | 206169 | 175244 | 87622 | 711 | 5 | 2 |
| 2 | Baja California Sur | 63730 | 56720 | 50481 | 25240 | 2401 | 12 | 3 |
| 3 | Baja California Sur | 774200 | 619360 | 495488 | 247744 | 4600 | 16 | 5 |
| 4 | Baja California Sur | 514036 | 436931 | 371391 | 185696 | 4470 | 9 | 3 |
| 5 | Chiapas | 797444 | 685802 | 589790 | 294895 | 3483 | 14 | 3 |
| 6 | Chiapas | 405064 | 340254 | 285813 | 142907 | 1725 | 5 | 2 |
| 7 | Colima | 776386 | 667692 | 574215 | 287108 | 2651 | 8 | 2 |
| 8 | Colima | 270730 | 235535 | 204916 | 102458 | 1276 | 5 | 3 |
| 9 | Colima | 265666 | 215189 | 174303 | 87152 | 1333 | 6 | 4 |
| 10 | Mexico City | 238756 | 198167 | 164479 | 82240 | 1399 | 5 | 3 |
| 11 | Guerrero | 621614 | 540804 | 470500 | 235250 | 2190 | 9 | 1 |
| 12 | Mexico State | 463504 | 393978 | 334882 | 167441 | 2708 | 13 | 1 |
| 13 | Michoacan | 556414 | 489644 | 430887 | 215444 | 2785 | 11 | 2 |
| 14 | Nuevo Leon | 694416 | 569421 | 466925 | 233463 | 4711 | 11 | 2 |
| 15 | Oaxaca | 340280 | 282432 | 234419 | 117209 | 2082 | 11 | 3 |
| 16 | Oaxaca | 536842 | 472421 | 415730 | 207865 | 3520 | 15 | 3 |
| 17 | Quintana Roo | 660062 | 547851 | 454717 | 227358 | 5228 | 17 | 3 |
| 18 | Sinaloa | 729816 | 623263 | 532266 | 266133 | 4166 | 8 | 3 |
| 19 | Tabasco | 689954 | 565762 | 463925 | 231963 | 2275 | 10 | 3 |
| 20 | Veracruz | 495530 | 424174 | 363093 | 181546 | 3279 | 8 | 2 |
| 21 | Veracruz | 648214 | 538018 | 446555 | 223277 | 3962 | 10 | 2 |
| 22 | Veracruz | 583714 | 478645 | 392489 | 196245 | 2750 | 9 | 2 |
| 23 | Yucatan | 479128 | 388094 | 314356 | 157178 | 1382 | 7 | 1 |
| 24 | Yucatan | 548152 | 465929 | 396040 | 198020 | 3385 | 14 | 1 |
| **Total** | | **12396204** | **10442256.74** | **8802902.687** | **4401451.344** | **68472** | **238** | **59** |

**Table S3.** Data set construction workflow information. Adapters and poor-quality reads (minimum Phred quality of 30) were removed using Trimmomatic software. Each data set was trimmed to a common length and the amplicon sequence variants (ASVs) within each pool were determined by clustering centroids with 100% identity using the VSEARCH software.

## Table S4

| Statistic | observed mean CI (95%) | null mean (CI 95%) | significance |
|---|---|---|---|
| **AI** | **5.6592 (4.7279-6.5381)** | **7.291 (7.0579-7.395)** | **< 0.001** |
| **PS** | **55.322 (53-57)** | **65.7873 (64.289-67.139)** | **< 0.001** |
| **MC (Baja California)** | 1 (1-1) | 1 (1-1) | 1 |
| **MC (BCS)** | **2.4316 (2-4)** | **1.0563 (1.001-1.1809)** | **0.01** |
| **MC (Chiapas)** | **1.9985 (1.998-2)** | **1.0063 (1-1.0139)** | **0.01** |
| **MC (Colima)** | **1.9987 (1.998-2.1)** | **1.0052 (1-1.015)** | **0.01** |
| **MC (Mexico City)** | 1.3261 (1-2) | 1.0393 (1-1.1371) | 1 |
| **MC (Guerrero)** | **1.9987 (1.998-2.1)** | **1.0052 (1-1.015)** | **0.01** |
| **MC (Mexico State)** | 1 (1-1) | 1 (1-1) | 1 |
| **MC (Michoacan)** | 1.4959 (1-3) | 1.0407 (1-1.1357) | 1 |
| **MC (Nuevo Leon)** | 1 (1-1) | 1 (1-1) | 1 |
| **MC (Oaxaca)** | **1.9985 (1.998-2)** | **1.0063 (1-1.0139)** | **0.01** |
| **MC (Quintana Roo)** | **1.9987 (1.998-2.1)** | **1.0052 (1-1.015)** | **0.01** |
| **MC (Sinaloa)** | 1.0127 (1-1.03) | 1.0149 (1-1.0149) | 1 |
| **MC (Tabasco)** | 1 (1-1) | 1 (1-1) | 1 |
| **MC (Veracruz)** | **1.9985 (1.998-2)** | **1.0063 (1-1.0139)** | **0.01** |
| **MC (Yucatan)** | 1 (1-1) | 1 (1-1) | 1 |
| **MC (Jalisco)** | 1.0431 (1-1.1) | 1.0315 (1-1.1481) | 1 |
| **MC (Tamaulipas)** | 1 (1-1) | 1 (1-1) | 1 |

**Table S4 supplement.** Phylogeny-trait association test. (AI, Association Index, and PS, Parsimony Score). Statistical analysis of the association between phylogeny and geographical locations of the sequence data.

**Figure S1**

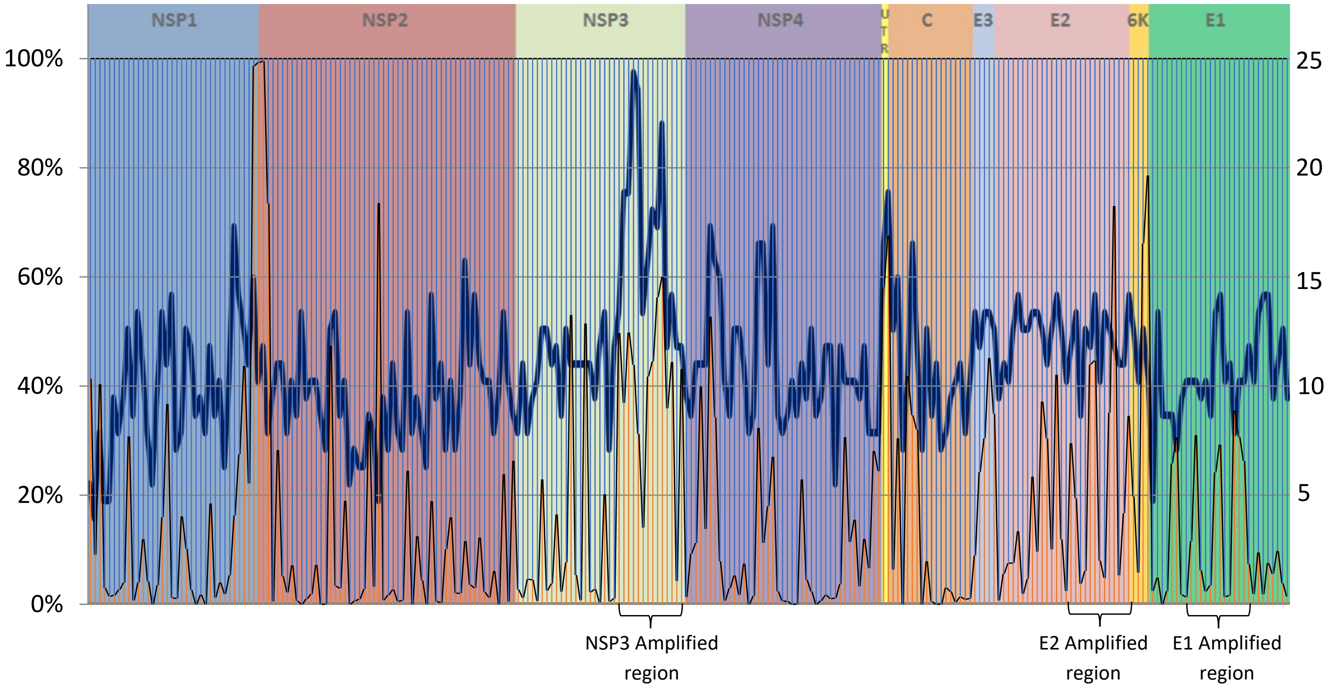


**Figure S1. Hypervariable sites identified** in 368 whole genome CHICKV sequences from the Virus Pathogen Database and Analysis Resource (ViPR) (NIH/DHHS) The "x" axis represents the position in the CHIKV CDS. Continuous blue line: Number of nucleotide changes in 45 nucleotides window (Right "y" axis). Red lines: Synonymous and non-synonymous mutation rate (Left "y" axis).

## Figure S2

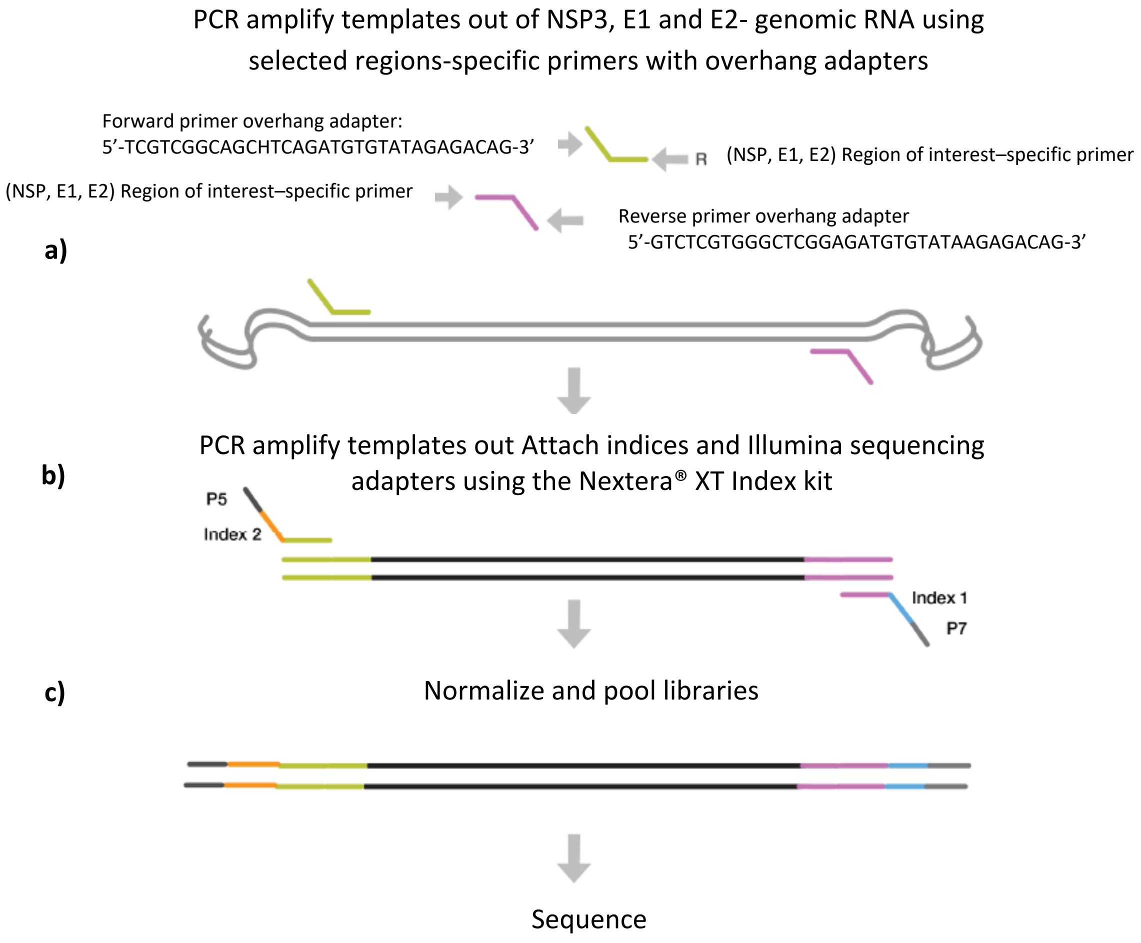


**Figure S2 supplement.** Amplicon workflow. **a)** Defined forward and reverse primers (Table S1) that are complementary upstream and downstream of the region of interest (NSP3, E1 and E2) were designed with overhang adapters and used to amplify templates from genomic RNA. **b)** A subsequent limited - cycle amplification step was performed to add multiplexing indices and Illumina sequencing adapters. **c)** Libraries were normalized, pooled and sequenced on the MiSeq system. Modified from reference [33].

**Figure S3**

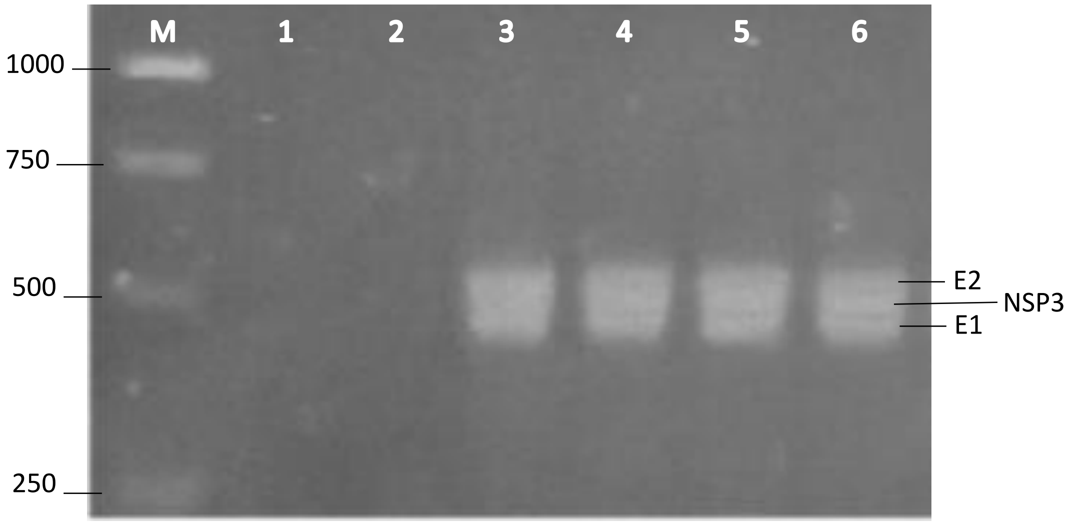


**Figure S3 supplement.** Agarose gel electrophoresis of PCR-amplified products from the CHIKV NSP3 (514 nt), E2 (547 nt) and E1 (479 nt) amplified regions in 4 of the serum samples selected for the study. Lane M = 1Kb ladder, lane 1 and 2 = negative control (distilled water). Lane 3 to 6 = RNA obtained from serum samples.

**Figure S4**

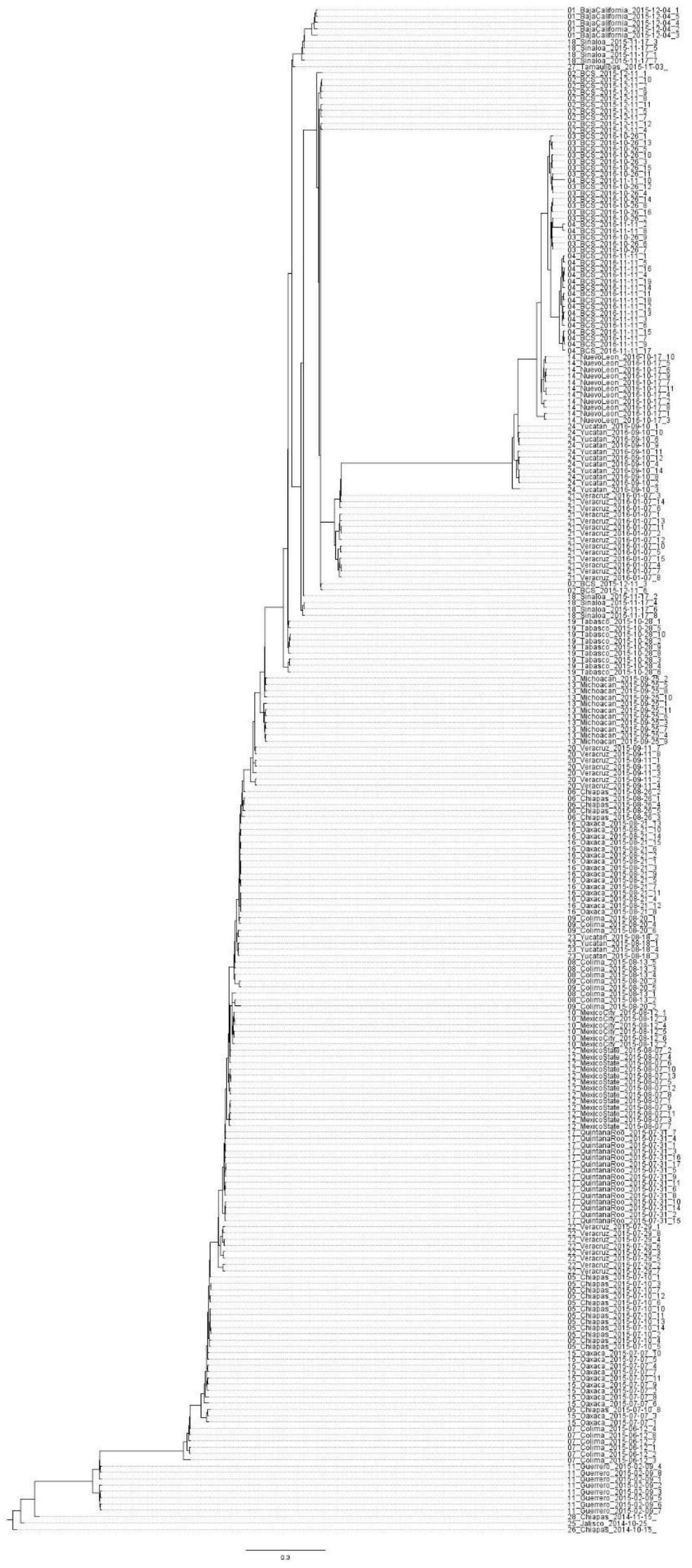

**Figure S4 supplement.** Preliminary MCC tree constructed with the 238 new sequences variants detected in our sequences.

**Figure S5**

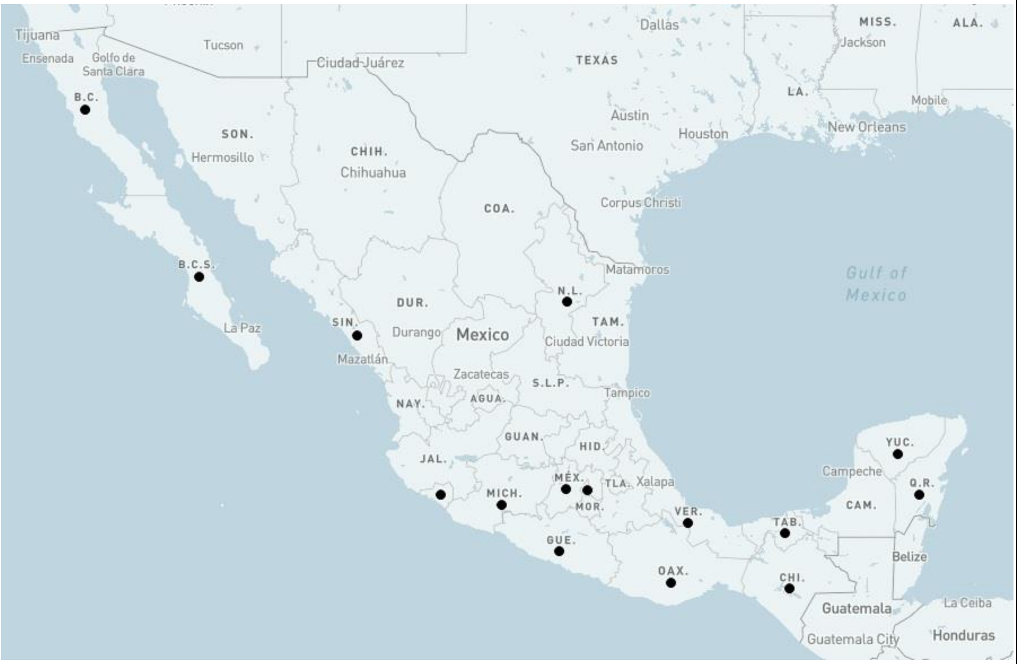


| Location | 2015 | 2016 |
|---|---|---|
| Baja California (B.C.) | 2 | |
| Baja California Sur (B.C.S.) | 3 | 8 |
| Chiapas (CHI.) | 5 | |
| Colima (COL.) | 9 | |
| Mexico City (cdMX) | 3 | |
| Guerrero (GUE.) | 1 | |
| Mexico State (MEX.) | 1 | |
| Michoacan (MICH.) | 2 | |
| Nuevo Leon (N.L.) | | 2 |
| Oaxaca (OAX.) | 6 | |
| Quintana Roo (Q.R.) | 3 | |
| Sinaloa (SIN.) | 3 | |
| Tabasco (TAB.) | 3 | |
| Veracruz (VER.) | 4 | 2 |
| Yucatan (YUC.) | 1 | 1 |

**Figure S5 supplement**. Left: Geographical distribution of CHIKV variant sequences used in this study. Black dots on the map indicate places where the original serum samples were obtained. Right: number of variant sequences obtained by location and year.

**Figure S6**

a)

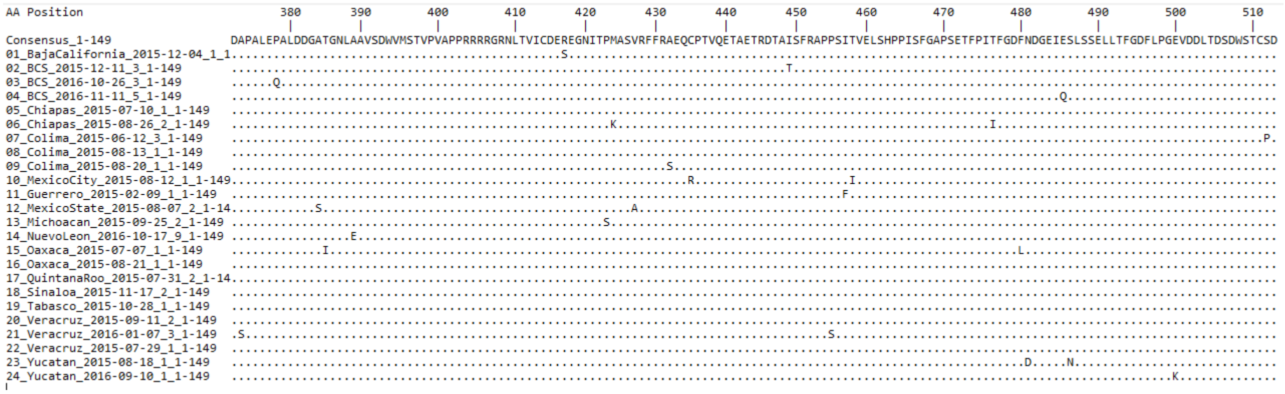

b)

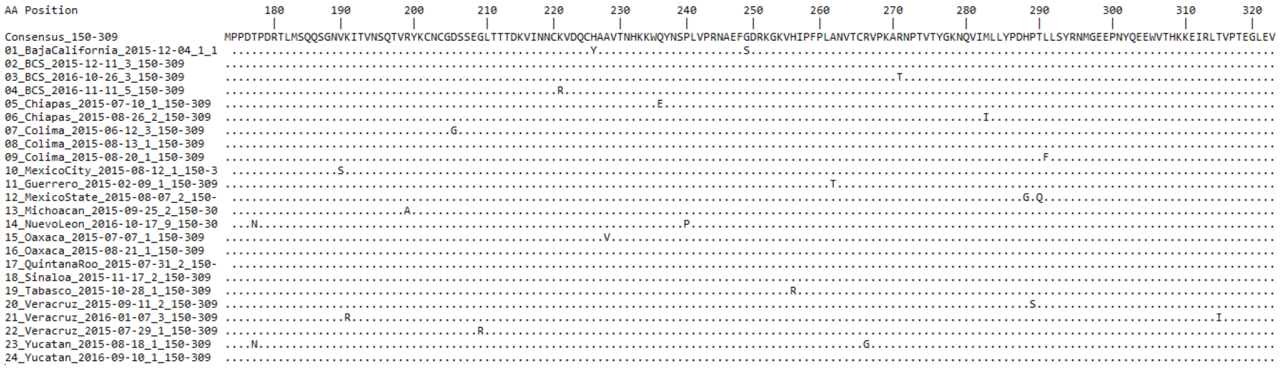

c)

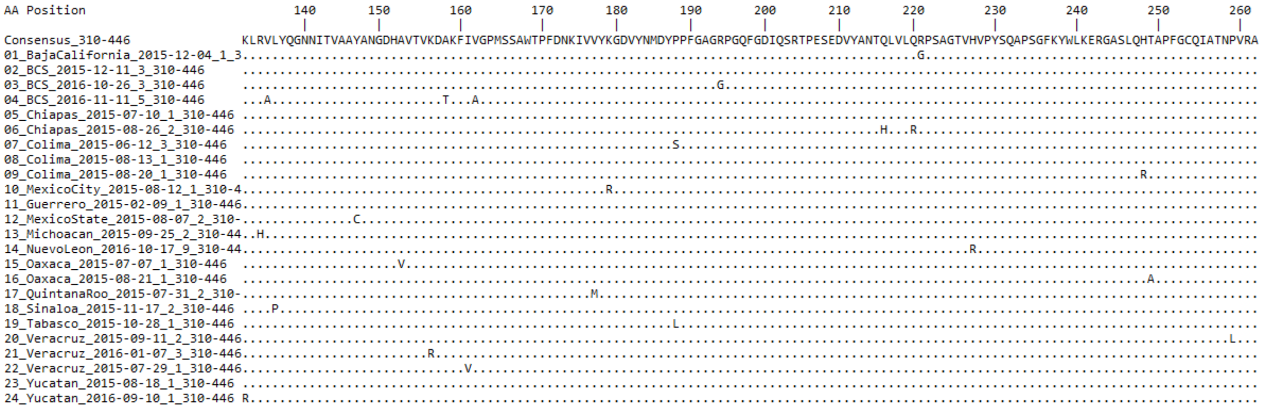

**Figure S6.** Non-synonymous mutations found in **a)** NSP3, **b)** E1 and **c)** E2. The first Mexican CHIKV sequence reported in October 2014 was used as the baseline sequence.
